# Evaluating User Preferences, Comprehension, and Trust in Apps for Environmental Health Hazards: Qualitative Case Study

**DOI:** 10.2196/38471

**Published:** 2022-12-22

**Authors:** Annabelle Workman, Fay H Johnston, Sharon L Campbell, Grant J Williamson, Chris Lucani, David M J S Bowman, Nick Cooling, Penelope J Jones

**Affiliations:** 1 Menzies Institute for Medical Research, University of Tasmania Hobart Australia; 2 Public Health Services, Tasmanian Department of Health Hobart Australia; 3 School of Natural Sciences, University of Tasmania Hobart Australia; 4 School of Medicine, University of Tasmania Hobart Australia

**Keywords:** health app, evaluation, air pollution, pollen, temperature, mobile phone

## Abstract

**Background:**

Climate change is projected to increase environmental health hazard risks through fire-related air pollution and increased airborne pollen levels. To protect vulnerable populations, it is imperative that evidence-based and accessible interventions are available. The environmental health app, AirRater, was developed in 2015 in Australia to provide information on multiple atmospheric health hazards in near real time. The app allows users to view local environmental conditions, and input and track their personal symptoms to enable behaviors that protect health in response to environmental hazards.

**Objective:**

This study aimed to develop insights into users’ perceptions of engagement, comprehension, and trust in AirRater to inform the future development of environmental health apps. Specifically, this study explored which AirRater features users engaged with, what additional features or functionality needs users felt they required, users’ self-perception of understanding app information, and their level of trust in the information provided.

**Methods:**

A total of 42 adult AirRater users were recruited from 3 locations in Australia to participate in semistructured interviews to capture location- or context-specific experiences. Participants were notified of the recruitment opportunity through multiple avenues including newsletter articles and social media. Informed consent was obtained before participation, and the participants were remunerated for their time and perspectives. A preinterview questionnaire collected data including age range, any preexisting conditions, and location (postcode). All participant data were deidentified. Interviews were recorded, transcribed, and analyzed using thematic analysis in NVivo 12 (QSR International).

**Results:**

Participants discussed app features and functionality, as well as their understanding of, and trust in, the information provided by the app. Most (26/42, 62%) participants used and valued visual environmental hazard features, especially maps, location settings, and hazard alerts. Most (33/42, 78%) found information in the app easy to understand and support their needs, irrespective of their self-reported literacy levels. Many (21/42, 50%) users reported that they did not question the accuracy of the data presented in the app. Suggested enhancements include the provision of meteorological information (eg, wind speed or direction, air pressure, UV rating, and humidity), functionality enhancements (eg, forecasting, additional alerts, and the inclusion of health advice), and clarification of existing information (eg, symptom triggers), including the capacity to download personal summary data for a specified period.

**Conclusions:**

Participants’ perspectives can inform the future development of environmental health apps. Specifically, participants’ insights support the identification of key elements for the optimal development of environmental health app design, including streamlining, capacity for users to customize, use of real time data, visual cues, credibility, and accuracy of data. The results also suggest that, in the future, iterative collaboration between developers, environmental agencies, and users will likely promote better functional design, user trust in the data, and ultimately better population health outcomes.

## Introduction

### Background

Climate change is predicted to increase health risks from environmental hazards worldwide. For example, climate projections suggest that many regions will experience an increased risk of air pollution from landscape fires [1], whereas exposure to heat-related health risks is projected to increase [2]. Adverse health outcomes associated with exposure to air pollution include lung and heart diseases, lung cancer, diabetes, neurological conditions, poor pregnancy outcomes, and premature death [3]. A changing climate is also likely to increase the health risks from airborne pollen owing to changes in pollen loads, allergenicity, and pollen season length [4]. The human and economic costs of such increases are likely to be substantial [5], and individuals living with pre-existing chronic conditions or from lower socioeconomic circumstances have been identified as the most vulnerable [6].

In this context, it is imperative to provide evidence-based, cost-effective interventions to reduce the impacts of environmental hazards on human health. Smartphone apps may offer part of the solution, for example, by providing individuals, including vulnerable populations, with easy and timely access to environmental hazard information or by providing tools to support the diagnosis and management of health outcomes triggered by environmental conditions [7-9]. Smartphone apps that collect symptom data can also provide a mechanism to track population-level health impacts of environmental hazards in real time [10]. For example, access to aggregated data on respiratory symptoms such as shortness of breath or heat-related symptoms such as light-headedness, can help public health departments monitor population health outcomes associated with environmental hazards and respond quickly to spikes in outcomes by releasing health alerts and targeted health campaigns [11].

Apps related to environmental health, such as air quality and pollen count apps, are rapidly proliferating and targeting different user groups, including children with asthma [12,13]. Although user perspectives and preferences regarding apps designed for other health conditions have been studied [14-16], there remains a paucity of evidence regarding how and why such apps do (or do not) support users in understanding and responding appropriately to atmospheric health hazard information. Some studies have investigated the effectiveness of environmental health apps with respect to messaging strategies [17] and behavior change support [18-20]; however, very few have tested user preferences related to app design or the extent to which users comprehend and trust the information presented. Given the plethora of possibilities available with respect to functionality, risk communication, and the design of both the overall app and individual features, it is imperative that we address this research gap to underpin effective and evidence-based environmental health app design in the future.

### Protecting Health From Environmental Hazard Risks

**Figure 1:**
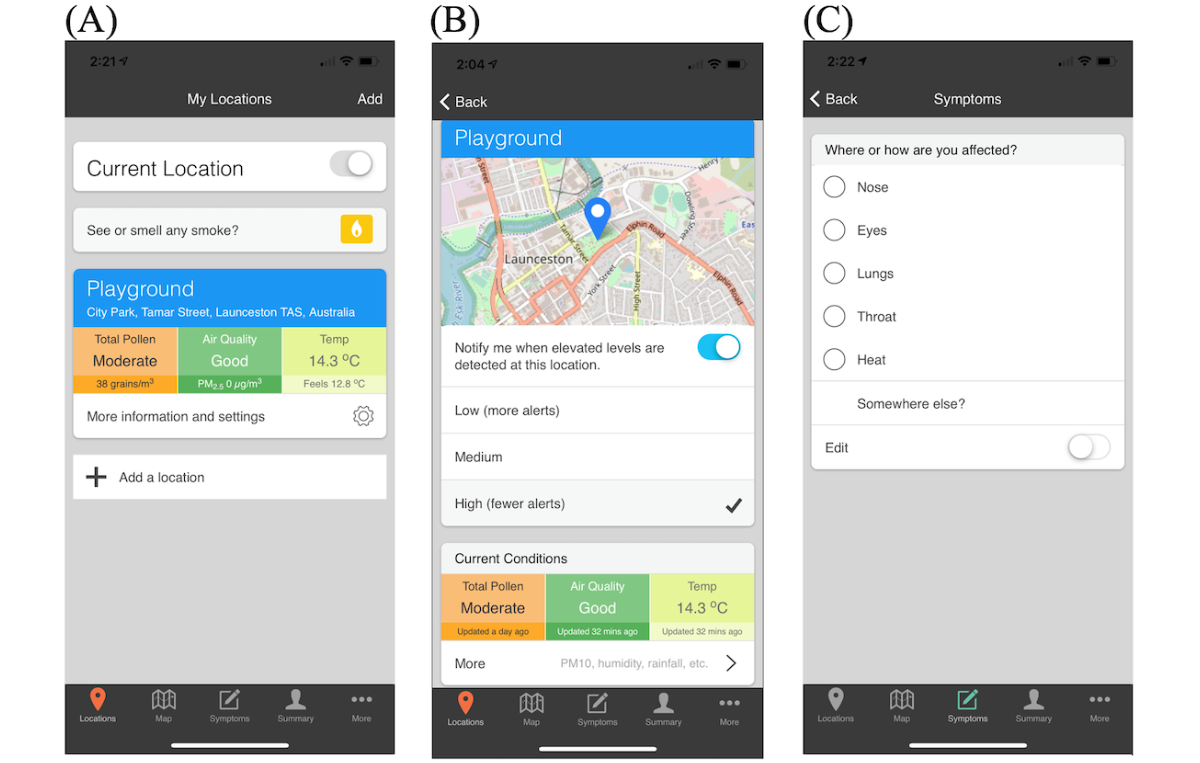
Key features and functionality of the AirRater app. Panel (A) shows the home screen where users can see up-to-date information on environmental conditions at their current or saved locations. Panel (B) shows how users can opt into alert notifications. Panel (C) shows part of the symptom reporting interface.

The AirRater app offers an ideal opportunity to explore these critical questions. AirRater was developed in Australia in 2015 with the aim of protecting individuals from 3 key atmospheric health hazards: pollen, particulate pollution, and extreme heat [8]. The app was co-designed by a consortium of multidisciplinary researchers and environmental and health government agency representatives and is now freely available across Australia. The functionality of the app has been described in detail elsewhere [8,19]; however, its core features include (1) the provision of near real time information on air quality, temperature, and pollen counts; (2) notifications when atmospheric conditions are poor; and (3) the capacity for individuals to log their symptoms and learn about their personal sensitivities (Figure 1)*.* Users can input and track symptoms related to the nose (eg, itchy), eyes (eg, watery), lungs (eg, wheezy), throat, and heat (eg, light-headed). Users can also input custom symptoms related to other health outcomes and self-report the severity of the symptoms experienced (mild, moderate, and severe). The symptom tracking functionality also enables the app to support public health surveillance and ongoing epidemiological research [21,22]. For a full outline of the app’s functionality and features, please see Multimedia Appendix 1. The app is supported by a website that provides users with information about data streams and sources.

### Evaluating User Preferences, Comprehension, and Trust

Several factors make AirRater an ideal case study to explore how users perceive and react to different features of environmental health apps and the extent to which users understand and trust the information presented. First, AirRater represents an unusually holistic environmental health app because it provides information on multiple environmental health hazards (pollen, air pollution, and temperature) in multiple ways (via maps and by saved location) and couples environmental hazard information provision with features designed to help individuals understand and manage their health conditions (eg, symptom tracking, symptom modeling, and personalized alerts). In addition, AirRater has been well-evaluated, and detailed information on who uses the app and their motivations for doing so has been previously published [19,23]. Importantly, these prior evaluations have also demonstrated that the app is successful in supporting a diverse range of users to make decisions and implement behaviors to protect their health, both in the context of severe air pollution episodes and more routine conditions [8,19,23].

This study leverages these characteristics and understandings of AirRater to explore how users perceive and respond to various features of environmental health app design and how such design features do or do not support user comprehension and trust in the information presented. Using qualitative methods, we explore user experiences with AirRater from this perspective, aiming to inform the development of effective environmental health apps. We specifically address 4 research questions.

1. Which features of the AirRater app do users engage with?
2. What are users’ additional information and functionality needs?
3. How do users perceive their comprehension of the information provided in the app?
4. What level of trust do users have in the information the app provides?

## Methods

### Overview

Numerous tools and frameworks exist to assess app integrity and quality, some of which have been validated [24-34]. Such tools are often prescriptive. Qualitative research methods provide the capacity to explore participant experiences in greater detail by enabling researchers to seek elaboration or clarification of initial participant responses. Accordingly, we developed a customized mixed methods evaluation framework based on the guide by the World Health Organization (WHO) to monitor and evaluate a digital health intervention [35] and the mobile app rating scale [34]. The WHO guide provides a practical framework and flexibility to explore specific questions of interest. The mobile app rating scale framework informed the thematic structure of the qualitative evaluation. On this basis, we developed a semistructured interview schedule that provided the capacity to prompt users for further details on their experiences based on their initial responses [36,37].

This study was conducted as part of a larger mixed methods study of AirRater users that covered multiple themes. The results with respect to usability and behavior change have been published elsewhere [19], and this paper specifically reports the results with respect to user perceptions of app design, comprehension, and trust.

In the interest of an independent evaluation, a qualitative researcher (AW) with no affiliation to the stakeholders involved in the app design and development was used to conduct the evaluation, undertake all interviews, and analyze all data. A qualitative researcher at the University of Tasmania, who was not affiliated with the app, reviewed the framework and protocol as an independent reviewer before its submission to ethics.

### Recruitment

As reported elsewhere [19], adults (18 years and older) were selected for recruitment if they resided in one of the following three locations in Australia: (1) Tasmania, (2) the Australian Capital Territory (ACT), or (3) Port Macquarie, New South Wales. Users were recruited from multiple locations to capture any location- or context-specific experiences, which were strategically chosen based on known episodes of high pollen days (Tasmania) and prolonged poor air quality because of the 2019-2020 wildfire events (ACT and Port Macquarie). Users were recruited through calls for participation via multiple media platforms, including a targeted email to registered AirRater users, an article in the AirRater newsletter, social media posts (Facebook and Twitter), and requests for participation during local radio interviews with AirRater team members. In total, 42 users were recruited to participate in the qualitative evaluation, with numbers almost evenly divided between Tasmania (20/42, 48%) and ACT (21/42, 50%). Despite multiple attempts to secure more participants, only 2% (1/42) of participants were recruited from Port Macquarie given that both locations experienced substantial wildfire smoke from 2019-20 wildfire events, ACT and Port Macquarie data were aggregated to protect the anonymity of the Port Macquarie participants.

### Ethical Considerations

An ethics application for the evaluation was submitted to and approved by the University of Tasmania Health and Human Research Ethics Committee (ID: H0015006). As per the ethics approval, participants who contacted the team expressing an interest in participating were sent an information sheet and consent form before confirming their eligibility. The information sheet included information on the purpose of the study, the study’s funding arrangements, details about what participants would be asked to do during the study, and the benefits and risks of participation. The participants were also informed that they were free to withdraw from the evaluation at any time. The evaluation and data collection methods were discussed with potential participants, who then returned their consent form if they were still happy to proceed with participation. Participants were notified that deidentified data would be stored at the University of Tasmania for a minimum of 5 years in accordance with the Australian National Health and Medical Research Council guidelines and would only be accessible to a subset of investigators involved in the evaluation (AW, FHJ, SLC, NC, and PJJ). Consent forms, as well as deidentified electronic transcripts and audio recordings, were stored in separate folders on a secure server at the Menzies Institute for Medical Research, Tasmania. All data were password-protected and accessible only to a subset of investigators involved in the evaluation (AW, FHJ, SLC, NC, and PJJ). Informed consent was obtained from each participant before their involvement in the study. Participants were remunerated for their time and contributions with an AUD $20 (US $14.50) gift card following their participation in the interview.

### Data Collection

Data were collected using 2 methods. First, a preinterview questionnaire delivered through SurveyMonkey collected key demographic details from the participants, including age range, any pre-existing conditions, and location (postcode, Multimedia Appendix 1). The questionnaire also collected data from participants on their personal descriptions of the app’s purpose, period of use, primary motivation for downloading the app, and perceptions of use over time. Second, semistructured interviews were then conducted. The full interview schedule was published in the study by Workman et al [19]; the interview schedule components relevant to the results presented in this paper are provided in Multimedia Appendix 1. All interviews were conducted via telephone or a web-based conferencing platform because of COVID-19 restrictions. With participants’ permission, all interviews were recorded to verify the accuracy of the transcripts.

### Data Analysis

Demographic data collected by the preinterview questionnaire were aggregated for descriptive analysis. The qualitative analysis software NVivo 12 (QSR International) was used to support thematic coding and analysis of the interview data [38]. All 42 interviews were transcribed by 2 research team members and verified by 1 before being uploaded to NVivo. Starting with the themes underpinning the interview schedule as an initial coding framework [39], all interview transcripts were thematically analyzed by the qualitative researcher leading the evaluation (AW). The overarching themes supported the identification of subthemes that emerged from the data. The results of the analysis were initially discussed with 2 researchers affiliated with the design and development of the app (PJJ and SLC) and who were involved in previous AirRater evaluations of user surveys.

## Results

### Questionnaire Data

The majority of the 42 participants (38/42, 90%) completed the preinterview questionnaire. Detailed results have been reported elsewhere [19]; however, the key results for contextualizing this study are presented in Table 1. Notably, most users had one or more respiratory conditions (asthma, allergic rhinitis, other lung conditions, or a combination), and most primarily used the app during seasons in which particular environmental triggers were present. Open-text responses from users indicated that they were most likely to use the app during the wildfire season (summer) or the pollen season (spring and summer), although some participants indicated that use was sporadic outside of these times.

**Table 1:** Summary of participant characteristics derived from the preinterview questionnaire^a^.

Characteristics		Responses, n (%)
**Sex**		
	Female	30 (79)
	Male	8 (21)
**Age range (years)**		
	21-30	3 (8)
	31-40	8 (21)
	41-50	4 (10)
	51-60	8 (21)
	61-70	9 (24)
	>70	6 (16)
**Pre-existing health conditions**		
	Asthma	17 (45)
	Lung condition other than asthma	9 (24)
	Allergic rhinitis	24 (63)
	Heart condition	3 (8)
	Stroke	0 (0)
	Diabetes	2 (5)
	Pregnancy	0 (0)
	Other	12 (32)
**Time since app download**		
	<6 months	8 (21)
	6-12 months	13 (34)
	1-2 years	6 (16)
	2-3 years	5 (13)
	3-4 years	5 (13)
	4-5 years	1 (3)

^a^Adapted from Workman et al [19].

### Interview Data

#### Overview

The interview results are reported below and stratified into four sections aligned with our research questions: (1) engagement with different features of AirRater, (2) additional information and functionality needs, (3) comprehension of AirRater, and (4) trust in AirRater. Additional interview data covering different themes have been published elsewhere [19]. This paper focuses only on the data relevant to the themes outlined above. The key findings relevant to these 4 themes are summarized in Table 2, and the remainder of this section presents detailed results from each theme.

**Table 2:** Summary of key findings from interview data, stratified by research question.

Research question	Summary of key findings
Which features of the app do users engage with?	The visual map and location features are used most frequently. Receipt of general alerts for elevated environmental hazard levels prompted app use. Participants used the symptom reporting feature to varying extents. A few participants indicated that the provision of real time information made AirRater their preferred information source.
What are users’ additional information or functionality needs?	Participants made a diverse range of suggestions and requests for additional data, features and functionality to enhance the app (see Multimedia Appendix 1 for a detailed summary). Common requests included a more detailed breakdown of pollen data, automatic notification of nearest monitoring station, the inclusion of wind speed or direction, and the capacity to download personal summary data for a specified period.
How do users perceive their comprehension of the information in the app?	Participants indicated they brought varying levels of scientific or air quality literacy to their interactions with the app. The interface design, such as the color code, 1-word rating system, helped participants and their family members understand environmental hazard data. Some participants indicated they were confused by the use of different metrics across different information sources.
Do users trust the information the app provides?	Many participants did not question the accuracy of environmental hazard data presented in the app. Some participants felt the app’s affiliation with a university provided credibility. Some participants expressed mistrust in AirRater data accuracy given their distance from a monitoring station. The extent to which app ratings aligned with personal symptoms and visual cues (such as visible smoke) influenced trust in AirRater data. Some participants questioned the conclusions of their personal health profile based on inputs to the symptom reporting feature.

#### Which Features of the App Do Users Engage With?

The participants were asked which features they used. Many participants (26/42, 62%) indicated using location (Figure 1 and Multimedia Appendix 1) and maps (Figure 1 and Multimedia Appendix 1) features most frequently. Some participants (4/42, 9%) specifically noted the value of the location function in supporting their decisions and monitoring the locations where they had a family:

I loved how it could have the multiple locations which for us over summer was really helpful cos we had family moving around...

ID_16, ACT

...one of the features I like was that I could set up... location zones and monitor not just how we were going but them (family) and use AirRater to determine whether they were likely to be having a good day or bad day and whether to give them a call or, you know if they would be OK today.

ID_10, ACT

I like it how you can... set your own locations and then it gives you... accurate data for the pollen level, air quality, and the temperature. And... the map feature so... you can look at sort of the air pollution levels on the map. I actually looked at that a lot during the bushfire season

ID_19, TAS

Importantly, the close-to–real time nature of the information available in the map and location functionality was particularly useful to people. A few (3/42, 7%) users indicated that up-to-date information was the core reason why they engaged not only with these specific features but with the app overall:

This one (AirRater) was far more useful from a day-to-day point of view than what we were getting information-wise from other sources...It didn’t help me knowing that we’d had the worst air in the world over the last 24 hours. What I needed to know was right now.

ID_34, ACT

...I ended up finding that AirRater seemed to have the most useful, up-to-date information... there was a lot of discussion amongst people and... I think that it ended up being that people started relying on AirRater being the most up-to-date, useful information.

ID_39, ACT

Some (7/42, 17%) participants indicated that the receipt of alerts for elevated environmental hazard levels (Multimedia Appendix 1) and reminders for symptom reporting were effective in prompting their engagement with the app:

I really like the notifications when it gave the particulate or the pollen rating cos then I double-check my medications and make sure I have done all the right things. Every time I was sent an alert, I would do a report....I liked the frequency, because I probably reported twice a week at least.

ID_24, TAS

...I would generally use it (symptom reporting) when I got a notification cos it would remind me to do it, and unless I’m reminded to do it then I tend to forget...

ID_28, TAS

There were varying levels of engagement with, and perspectives on, the symptom reporting feature more generally (for an illustration of the symptom reporting feature, see Multimedia Appendix 1). Some (4/42, 9%) participants liked the feature and found it easy to use:

I liked it actually. I just clicked on mild, moderate...and I thought, oh god, I’m a bit of whinger...then I thought, no, this data will be good for somebody, and I’ve just found now the summary, which is really good

ID_38, ACT

...the reporting symptoms feature works really well... prompting you to report symptoms and report medication taken...[ID_41, ACT]

However, some (9/42, 21%) participants identified technical and practical issues with the symptom-reporting feature:

...there was no feedback to say, you know, your symptoms have been constant for the last week, you should go and see a doctor or anything like that. It was literally...just answering the questions...do you have itchy eyes, what have you done, have you put eyedrops in or whatever, so I’m just ticking the things that I have to tick and then that was it, now I’ve done my homework (laughs) That’s how I saw it.

ID_35, ACT

...Most of the time I have symptoms, and most of the time my symptoms are controlled by medication. The way this works, I have to tell them I’ve got a runny nose...and it’s mild. Why is it mild? Because I’m on medication...when you’re doing this, you know, four or five times a day you do get sick of that kind of stuff

ID_13, TAS

#### What Are Users’ Additional Information and Functionality Needs?

Participants were asked whether there were any additional features or information they wanted AirRater to provide. Most (38/42, 90%) participants recommended at least one enhancement to the app to supplement existing features or requested the provision of additional information. Given the diversity of participants, their specific health conditions, and their unique information needs, the responses were wide-ranging. However, common requests included a more detailed breakdown of the pollen count data, automatic notification of the nearest monitoring station, inclusion of wind speed or direction, and the capacity to download personal summary data for a specified period. A full summary is provided in Multimedia Appendix 1; examples of responses included the following:

...the total pollen count’s irrelevant...why should I care...if there’s a lot of gum tree pollen around? ...I’m not allergic to gum tree pollen...I want to know specific levels of specific pollens which apply to me

ID_13, TAS

I understand now why the PM2.5 is the most obvious particle size that’s there (for air quality) so I sort of understand the other one (PM10) being a bit subliminal... I have found that I’ve had to hunt for, I’ve had to discover it for myself rather than it all being presented there on the front page...

ID_27, TAS

...it would be good if they’d (AirRater) just tell me where the closest station is... getting the information from... so the user knows how accurate it is

ID_2, TAS

I find windy conditions exacerbate my asthma...so even just having a wind speed...I feel like wind speed is more important than temperature...

ID_7, TAS

What I would like to see is the capacity to be able to print it out... over a period of time, it would be perhaps better to look at it that way... and find out what the changes were.

ID_37, ACT

#### How Do Users Perceive Their Comprehension of the Information in the App?

Participants were asked to describe their understanding of the information the app provided, how the interface does or does not support their comprehension, and whether additional information is needed to assist them in understanding the data. Participants indicated varying levels of perceived general air quality literacy; however, irrespective of this, most (33/42, 78%) participants indicated that they were able to sufficiently understand the information in the app to support their needs:

I have a very good understanding of the...information. I have a science degree... so I don’t have any trouble understanding the information and I know exactly what the particulate issues are and all the rest of it.

ID_4, ACT

...every now and then I thought, that’s interesting that that’s the metric for good and that’s the metric for poor...because the poor kicked in at quite a low numeric value...and more just a curiosity cos I didn’t know enough about...the scale that’s used for air quality. ...if I got really seriously curious I could have Googled what the Australian air quality standards meant, but...I was happy to take, that’s the standard, that’s the number, I can make a decision on that.

ID_5, ACT

I knew nothing about air quality data before the bushfires...I know that there’s...a website associated with it (AirRater) where the data is translated from and...I did look at that a few times to sort of try and...get an idea of...what does this PM2.5 thing mean...I got a fairly basic but comprehensive understanding of the rating system in terms of what it meant when it said ‘good’ or ‘poor’...and what that might mean for my health, but I certainly wouldn’t ever be able to like tell somebody else it in a comprehensive manner. I just learnt enough to know that I felt comfortable that what was happening in the app was a reflection of comprehensive and good data that I could trust.

ID_14, ACT

I had to Google what PM squared meant, in air quality, and I still didn’t quite get it, but um, then I just look at the word good now, or poor, or whatever. I didn’t understand that much, um, and then I thought, oh well, that’s fairly scientific actually which is good, but yeah, I didn’t understand, and I s’pose I don’t really need to understand that, I just Googled it out of interest.

ID_38, ACT

Numerous (9/42, 21%) participants indicated that the interface helped them and their family members comprehend the information.

...some of my family members...they’re from non-scientific backgrounds...they didn’t seem to understand it...as well as me, but they could still get a sense of...what the information was showing, so I think it’s good that, like you have color codes for the different levels.

ID_19, TAS

I think the interface is fairly user-friendly for people who don’t necessarily have...the science and health background to...interpret it, because of the color change and the...good, fair, poor, that made it really quick and simple to understand.

ID_34, ACT

...I really liked especially for my kids being able to explain the coloring. ...my eldest is like, oh it’s red, we can’t go outside. Oh, it’s purple, no, don’t even open the window...

ID_16, ACT

...it became a bit of a family game...we had different levels of how many trees we could see outside (laughs) ...we actually started being able to have a good gauge of...what the numbers, levels were like, and...connecting those to our visual observations, so...it became quite an interest factor as well for the whole family.

ID_34, ACT

Several (6/42, 14%) participants discussed how the use of different air quality metrics on different apps and websites affected their own or others’ ability to understand air quality information (in AirRater and beyond). This was particularly relevant from the beginning of 2019 to 2020 wildfire events because of the diversity of air quality metrics used by various sources:

...there was a wide disconnect in the greater community...because different people were using different pieces of information. So, the ones who were using 24-hour rolling...worked on a different poor/fair scale than the AirRater scale...

ID_34, ACT

...people were using...a bunch of different apps that all had really different information...and people were really confused and they’re like, well, this is telling me...everything’s fine and that I can go outside, and I’m like, no, it’s cos that’s an average...

ID_39, ACT

#### Do Users Trust the Information the App Provides?

Participants were asked whether they had ever questioned the data provided by AirRater. Participants indicated varying levels of trust in the information provided by AirRater. Many (21/42, 50%) participants did not question the accuracy of the data, citing reasons such as their inclusion on credible government agency websites and the comprehensiveness of the supplementary information.

I did read like, where it was made, and who was running it and all those bits, and it was also helpful, like, that it was on the ACT Health website as well, I think.

ID_31, ACT

...I had read a fair amount about it (air quality)...just out of interest because I do try and keep up with what’s going on out there...When it came out on the (ACT government) health website...I thought, well... that’s good enough.

ID_37, ACT

I found that the one (app) that I implicitly trusted the most was just the AirRater one, because the information on the website was so comprehensive about, like this is how we are measuring it and this is why it’s a good way of measuring it, and...it wouldn’t be inaccessible to people without that sort of background and knowledge, but it was also like, it was also this is good data because of this reason, so it was enough information to go, yep this is what I trust, this is what I am okay with despite these debates happening in these social media spaces that I was involved in.

ID_14, ACT

Some (3/42, 7%) participants acknowledged that their trust in the information—and in the case of one participant, trust in sharing personal information—was a result of their affiliation with a university, with references to the importance of data protection and ethical integrity:

...because it was run by a university...I implicitly trust...with data protection approaches and stuff...a university, particularly in Australia, with the really stringent ethics requirements that we have...I do feel comfortable sharing my data and sharing my information...that was a huge...reason why I even downloaded the app in the first place.

ID_14, ACT

...*if it’s associated with a university it’s more likely to be neutral, less likely to be contaminated by advertising dollars... generally it just seemed to me that it’s likely that whichever algorithm you use or whatever to deduce it, it’s probably likely to be quite accurate*...[ID_15, TAS]

In contrast, several (10/42, 24%) participants indicated that they had questioned the information presented, in many cases attributing this lack of trust to a lack of data sensitivity or accuracy away from the monitoring stations:

I felt like the location, the air quality...wasn’t necessarily sensitive enough, I think also because I live in a valley, so...I might be...experiencing something at my home that wasn’t showing up on...the map at all...

ID_1, TAS

...the air monitors don’t actually accurately monitor where I am...we’re just in a bit of a black hole...it’s really limited, and I sort of stopped using it (AirRater) a lot because it was just, the data isn’t there for what I was most needing it for.

ID_23, TAS

...one of the issues even across an area the size of Canberra is the number of monitoring stations, so we’re always taking AirRater and even the ACT air quality index with a grain of salt compared to your actual local situation.

ID_10, ACT

I think I noticed that our closest one was Belconnen, which is probably not too far away... 10 or 15 kilometers away, so I guess you just sort of like, take that a bit into consideration. (ID_20, ACT).

The importance of personal experience with symptoms, as well as (the absence of) visual cues, also proved a fundamental factor influencing user trust, as some (3/42, 7%) participants acknowledged that they were at times skeptical or surprised by AirRater information:

Sometimes I’m feeling really rubbish and I look...it says that the pollen count’s low, and I’m like, oh well then, maybe I’m not well, but if you go and look at a grass count specifically it says that it’s quite high and like, oh that’s contradictory...

ID_21, ACT

you’d look outside and think, oh wow, I can see sort of the haze in the background but, I’ve...got close to normal...visual range, it doesn’t look bad, it doesn’t smell bad, but also realising that...harmful air quality kicks in the level below what the normal human can sense so, I tended to say, OK, yeah, if it’s telling me that (air quality is poor), it just means I can’t see the bad stuff, or smell the bad stuff.

ID_5, ACT

A couple of times... I’ve felt a bit tight in the chest and I’ve been surprised that the air quality’s been good... but... I put it down to there must be something else going on in my body that was causing that tightness, and it wasn’t the air quality.

ID_6, TAS

Some (3/42, 7%) participants also queried the accuracy of the conclusions in their personal health profile based on inputs into the symptom reporting feature.

AirRater was saying...your problem is with animals, because you’ve (been) exposed to animals and you’re having symptoms, and I was like, well, actually it’s not that simple, it’s not that clear cut, and I’ve had animals for longer than I’ve had symptoms, so it’s not necessarily the case...

ID_1, TAS

...sometimes...I had a look at the graph when I did my symptoms and...it was all mornings and I’m like, pretty sure I said 24 hours for some of those, so I’m not sure if it’s captured that as well as it could’ve.

ID_31, ACT

## Discussion

### Principal Findings

#### Overview

Through semistructured interviews with 42 users, this study gained valuable insights into what features are useful, what facilitates user comprehension, and what influences users’ levels of trust in the information provided by AirRater. Specifically, we found that location and map functions are the most useful features of AirRater for users. Furthermore, most participants were able to suggest app enhancements based on personal needs or preferences (Multimedia Appendix 1). We also found that, irrespective of self-reported literacy levels, most participants reported that the information provided in the app was easy to understand and supported their needs. Finally, we found that many participants did not question the accuracy of the data presented in the app. As discussed below, this new knowledge extends our understanding of effective design principles in environmental health apps and highlights key considerations for future app design (Multimedia Appendix 1). Furthermore, our results confirm that AirRater is used by a diverse group of individuals with unique health conditions, personal preferences, and information needs, highlighting the need for environmental health apps to manage the core design tension between meeting diverse user needs and streamlining for ease of use and comprehension. In the 4 sections below, we discuss our key findings in further detail and compare them with those of previous research. We also discuss implications for the development of future environmental health apps.

#### Engagement With Features

The map and location features of AirRater were highly valued because they supported users in monitoring the movement of hazards or environmental conditions in other locations where family members were based. This finding is pivotal for informing the future design of environmental health apps, as the ability to access hazard information quickly and easily at multiple locations supports app users not only to assess, plan, and mitigate their own risk but also that of family members located elsewhere. Furthermore, numerous participants reported that the provision of near real time data via the map and location functions was highly valued, particularly in the context of rapid changes in air quality during the Australian 2019-2020 wildfire seasons. Other studies have determined the importance of accurate and timely information. For example, Gooze et al [40] found that consumer satisfaction and use increased following the introduction of real time information for a transportation information tool. For environmental hazard information in particular, the presentation of near real time information is critical for users to adequately support decision-making.

We also found that alerts for hazard levels and symptom reporting were effective in prompting users to look at and engage with AirRater. This finding is congruent with earlier research findings of increased engagement and higher-frequency app visits from push notifications [18,41]. The role of alerts in hazard communication is particularly important for “invisible” threats, such as pollen and, at times, air quality, as it can raise awareness and literacy of such hazards. Finally, AirRater participant perspectives on the symptom reporting feature varied; a few participants indicated that it was useful, while other participants indicated that they had experienced both technical and practical difficulties with the feature. Difficulties reported included the inability to modify symptom reports and the repetitiveness of symptom reporting. Previous research findings have confirmed that repetitive and multistep processes can discourage users from engaging with a particular app or feature. In their analysis, Cho et al [42] examined cognitive factors, including eHealth literacy and health app use efficacy, in app use among a sample of 765 app users from South Korea. They found that health app use efficacy is correlated with continued app use and hypothesized that app features that require multiple steps to access information require users to invest time and energy, which may impact their perceived health app use efficacy [42]. Our findings underline the importance of simplified and streamlined symptom reporting processes to maximize the engagement and utility of such features in environmental health apps.

#### Additional Information and Functionality Needs

As detailed in the Multimedia Appendix 1, participants suggested numerous enhancements to the app, including the incorporation of additional meteorological information (eg, wind speed or direction, air pressure, UV rating, humidity), functionality enhancements (eg, forecasting functionality, additional alerts, the inclusion of health advice), and clarification of existing information (eg, symptom triggers). Four common requests were presented by the participants: (1) a more detailed breakdown of pollen data, (2) automatic notification of the nearest monitoring station, (3) the inclusion of local wind speed or direction, and (4) the capacity to download personal summary data for a specified period. Our results on additional information and functionality highlight that AirRater users are diverse in their personal information and functionality needs. Although this diversity demonstrates the capacity of an environmental health app to effectively engage various populations, it also implies the fundamental importance of app personalization and customization to meet individual user needs [43,44], particularly when balancing the simultaneous need for simplicity and streamlining to enhance usability and comprehension.

#### Comprehension

First, despite acknowledging that they brought varying levels of air quality literacy to their engagement with AirRater, participants indicated that the current AirRater design, particularly the use of colors, facilitated their understanding of the data. The relationship between interface design and information comprehension has been reported by other researchers. For example, Caburnay et al [45] assessed a random sample of 110 diabetes-related apps for health literate design. They found that most apps studied used enabling elements, such as everyday language (88/110, 80%), bold and contrasting colors (89/110, 80.9%), and included visual, customizable content (77/110, 70%). Similarly, in their study exploring stakeholder perspectives for improving storm surge risk communication, Morrow et al [46] found that stakeholders preferred maps that used multiple colors to convey different levels of storm surge risk. Our results reaffirm the importance of color as a part of a strategic design approach for environmental health app development. Another key result was that some participants indicated that the use of different indices and metrics across information sources was confusing during the wildfire season (eg, 24-hour average vs hourly or real time levels). In the absence of standardized information, our findings highlight the importance of providing clear and accessible information on how to interpret the specific metrics presented for a given setting and the value of supplementary information via multiple media to address confusion when it arises. Finally, Stonbraker et al [47] found a group of end users with low health literacy preferred simple bar graphs with emojis, again emphasizing the need for simple visualizations that convey the health implications of data. Stonbraker, Porras, and Schnall [47] also emphasized the importance of testing visualizations with target groups, a targeted approach that could be used in future iterations of AirRater to assess preferences and verify comprehension.

#### Trust

Many participants reported general trust in the information provided by the app, without questioning the accuracy of the information provided, with some reporting AirRater’s affiliation with a university as a pivotal factor. Our findings are congruent with results reported elsewhere [48]. For example, in their scoping review on trust in digital health interventions, Adjekum et al [49] identified factors such as ease of use, self-efficacy, customizability, credibility, and stakeholder engagement as enabling trust in digital health interventions. Participants who expressed mistrust in the information provided by AirRater reported distance from an air quality monitor or the app’s conclusions about their personal triggers as their reasons to question the data, although this did not seem to impact their overall willingness to use the app. This finding underscores the importance of data integrity in environmental health apps for protecting individuals from unnecessary or inadvertent exposure to environmental hazards. The need for a comprehensive and accurate air quality and pollen monitoring network is pivotal to ensuring that environmental health apps can effectively support populations. It also suggests opportunities exist for indoor air quality monitoring and the potential for environmental health apps to connect with devices, such as indoor air quality monitors or air purifiers, to provide supplementary data on potential triggers present in an indoor environment.

#### Implications for Future App Design and Future Research

**Figure 2:**
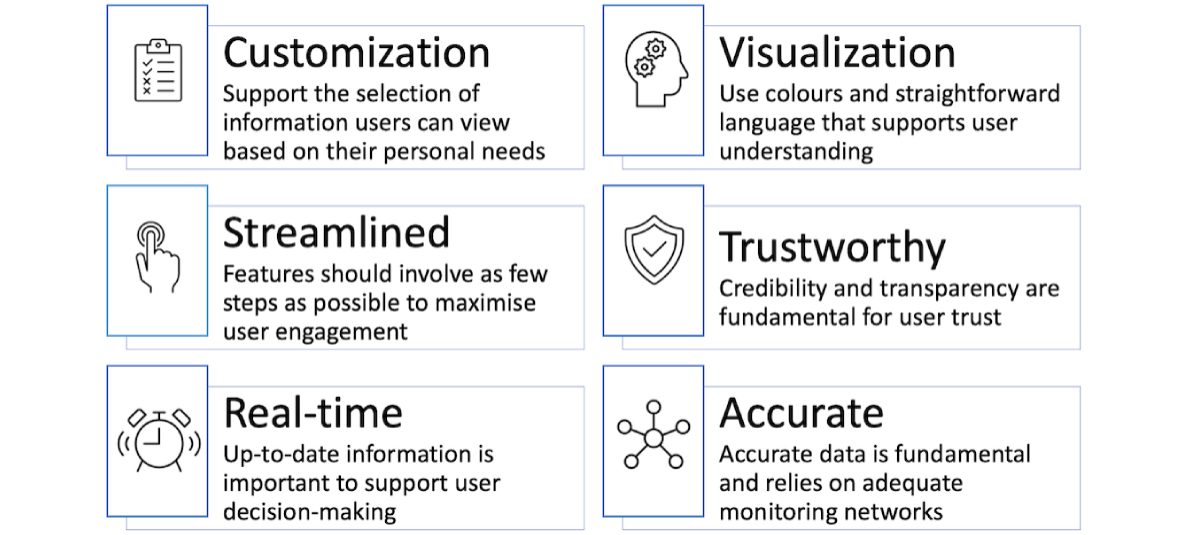
Implications for future environmental health app design.

Figure 2 synthesizes the core implications of our study with respect to the environmental health app design. First, our study clearly showed that users value a level of customization with the capacity to access and select information based on their individual needs. However, streamlined design is important: users prefer features that are visual and involve minimal steps. In addition, users value real time information with transparency around data sources. Our results also highlight the importance of using visual cues to support comprehension; for example, the use of a one-word color rating system in AirRater supports users in understanding scientific information irrespective of their perceived level of health or air quality literacy and to support their family members, including children. The credibility of the app developer also proved to be important for influencing user trust as well as the perceived accuracy of data, in this case, provided by established air quality and pollen monitoring networks. Accordingly, environmental health apps must be supported by sufficient data inputs from adequate and robust monitoring networks. To ensure that key elements are integrated into app design, iterative collaboration among developers, environmental agencies, and users is likely to support better app functionality, enhance user trust in the data presented, and support the ultimate goal of improving population health outcomes.

With respect to future research, this evaluation of AirRater as an example of an environmental health app suggests several future research needs. First, as environmental health issues can be highly localized, there is merit in pursuing similar evaluations of app utility and efficacy in other jurisdictions and countries. Although AirRater is currently only available in Australia, the implementation and evaluation of environmental health apps, such as AirRater, in other settings would extend the conclusions outlined here. Second, there is a strong need to investigate usability and comprehension among a more diverse range of user groups, including caregivers, children, and the elderly, across environmental health apps, to establish specific preferences and needs and to optimize health outcomes. At last, with respect to trust, it is important to investigate whether levels of trust in app data change because of likely changes to the air quality and pollen monitoring network in the coming years, either through the expansion of the existing network or the introduction of low-cost air quality sensors for individual residential use.

### Limitations

Several limitations impact the strength of the conclusions presented in this study. First, in sampling a cohort of current AirRater users, results presented here are most useful for the purposes, as intended, of an in-depth evaluation of AirRater. However, the sample is not likely to be representative of all AirRater users or other populations who may choose to use environmental health apps. In this context, it is not possible to determine the extent to which the results presented here are generalizable to other groups. None of the participants in this study were culturally and linguistically diverse. Our choice of a “within person” study design has been vindicated by recent digital health reviews however greater diversity of participants would have been useful [50]. Another recognized limitation is the lack of representation of older children and young adults. Similarly, while a small number of respondents indicated having children aged under 15 years, conversations focused on the personal use of and experience with the app. An opportunity exists to evaluate AirRater further by targeting older children (ie, 10-17–year-olds) and young adults (ie, 18-24–year-olds) to determine whether any age-specific customization is required to support further uptake of the app in these age groups. This is particularly important, given the app’s educational potential, as indicated by some participants. Finally, this evaluation only captures insights from AirRater app users, who are both engaged with the app and agree to participate. The opinions and responses of historical and current AirRater app users disengaged from the app were not accounted for in this study. Actively seeking the perspectives of users who no longer engage with the app would prove valuable for gaining unique insights into potential barriers to app use, as well as additional suggested app enhancements.

### Conclusions

This qualitative evaluation of the free smartphone health app AirRater explored user preferences, comprehension, and trust. The participants’ perspectives can inform the future development of environmental health apps. Accordingly, the perspectives presented in this paper contribute to identifying key considerations for successful environmental health app design, including customization, streamlining, use of real time data, visual cues, credibility, and accuracy of the data. These considerations are more likely to be prioritized when apps are designed in collaboration with developers, environmental agencies, and users. In the future, environmental health apps are likely to play a pivotal role in supporting populations facing increasingly severe and frequent global environmental changes and extreme events. Ensuring that environmental health apps are fit for purpose, effective, accurate, comprehensible, and trustworthy will assist in maximizing the health outcomes of the population.

## Appendix

Multimedia Appendix 1 A summary of AirRater features and functionality, interview questions and suggested app enhancements.

## Abbreviations

ACT: Australian Capital Territory
WHO: World Health Organization

## Data Availability

The data sets generated and analyzed during this study are available from the corresponding author upon reasonable request.
