# Supplementary material for: Evaluating User Preferences, Comprehension, and Trust in Apps for Environmental Health Hazards: Qualitative Case Study
Source: JMIR Form Res. 2022 Dec 22;6(12):e38471. doi: 10.2196/38471 (PMC9816954; doi:10.2196/38471)
Supplement: Multimedia Appendix 1 [file formative_v6i12e38471_app1.pdf]

Supplementary Material

Table S1. Summary of AirRater features and functionality

**Feature/ functionality**	**Details**
Environmental hazard information by location	an interface presenting data on three key environmental hazards – air quality (measured by particulate matter, PM), temperature and pollen counts – and sources near real-time data directly from relevant government agencies (see **Figure 1a.** in manuscript and **Figure S1** in Supplementary Material).<br>These data can be accessed in two ways – via a home screen showing the data at the user's current or saved locations, or via an interactive map (see below). The data for the app are sourced from federal and state-based agencies, including the Bureau of Meteorology for temperature data, state-based agencies that control the air quality monitoring network in their respective jurisdiction for air quality data, and select research institutes for pollen data. When the app is used in a location more than 20 kilometers from a monitoring station, the European Union's Copernicus Atmosphere Monitoring Service (CAMS) global model is used to estimate air quality.
Hazard rating	a color-coded, word rating system that is presented with the environmental hazard data. For example, the app indicates the air quality is 'Fairly good' with a yellow background when airborne particles (measured as $PM_{2.5}$) are between 10-24 mg/m^3 (see **Figure 1a.** in manuscript and **Figure S1** in Supplementary Material). Where a user is more than 20 kilometers beyond a monitoring station, the app uses a different color-coded scale to distinguish that air quality is estimated.
Interactive map	an interactive map of Australia allows users to see environmental data at 'pin drop' locations and/or view several map overlays, including monitoring station locations, symptom hotspots and planned burns (see **Figure 1c.** in manuscript and **Figure S2** in Supplementary Material).
Symptom reporting	a feature that supports users to capture their symptoms and symptom severity, medication use and potential additional triggers that may be present (e.g. dust and animals) at the time symptoms are experienced (see **Figure S3** in Supplementary Material).
Personal exposure summary and symptom analysis	a personalized dashboard summarizing historical symptom data alongside historical environmental hazard data, to visually demonstrate any relationships between symptoms, potential triggers and environmental hazards.
General and personalized alerts	an option to create and receive general alerts for low, medium or high elevated levels of air pollution and pollen counts. If users input sufficient symptom data over time, they establish a personal trigger profile which allows them to create and receive personalized alerts (see **Figure 1b.** in manuscript and **Figure S4** in Supplementary Material).

Figure S1. Presentation of the environmental hazard information by location function in the AirRater app

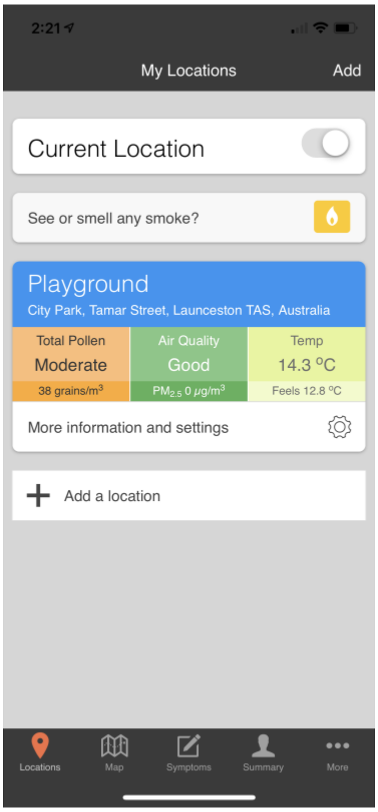

Figure S2. Presentation of the interactive map function and overlay options in the AirRater app

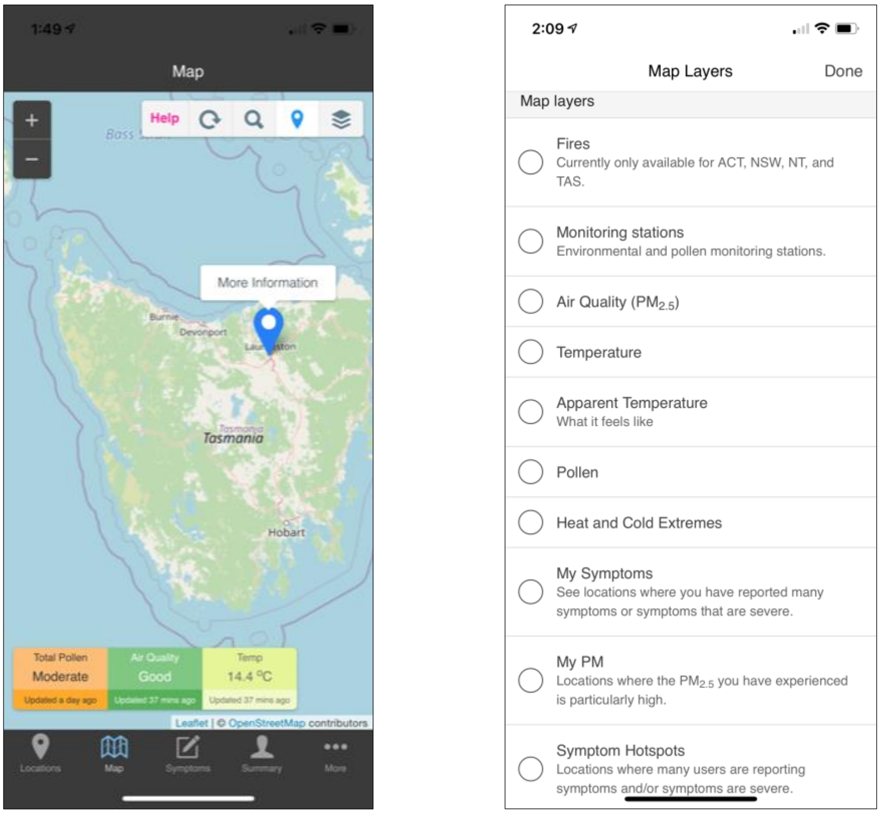

Figure S3. Presentation of the symptom reporting functionality in the AirRater app

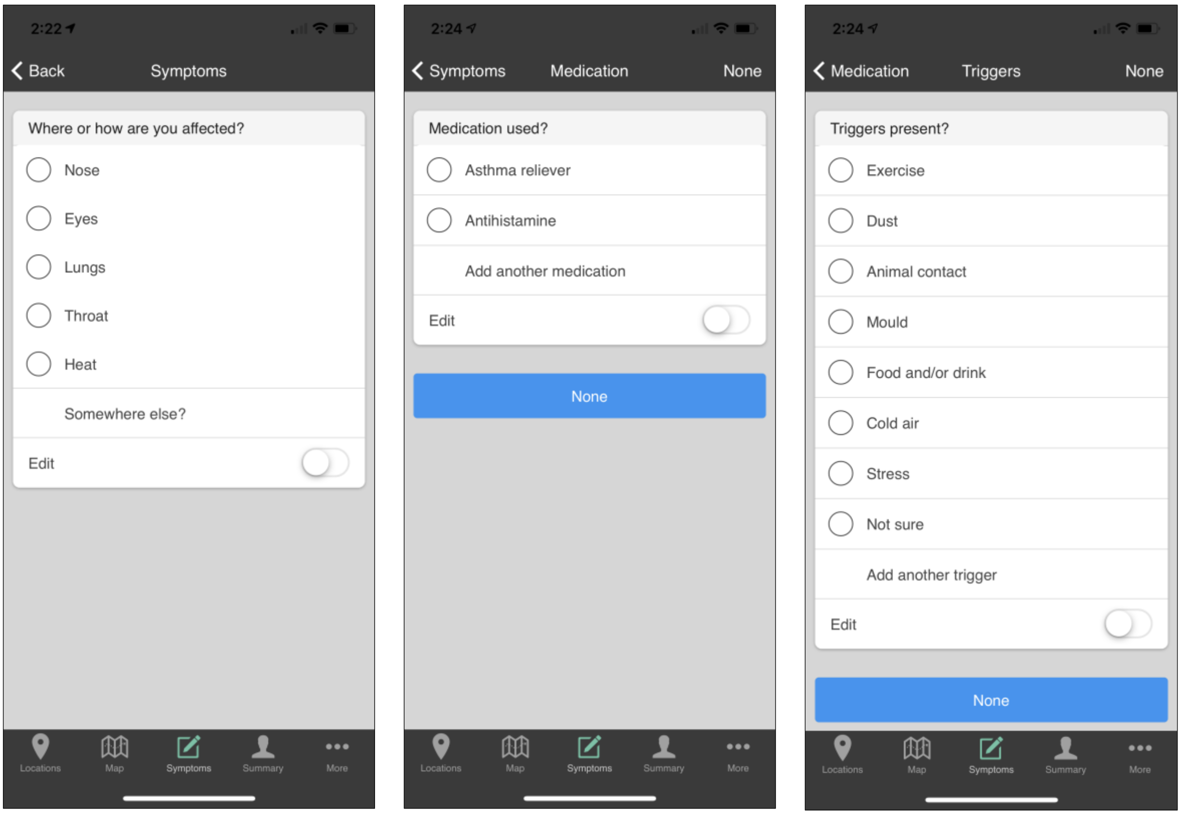

Figure S4. Presentation of the general alerts functionality in the AirRater app

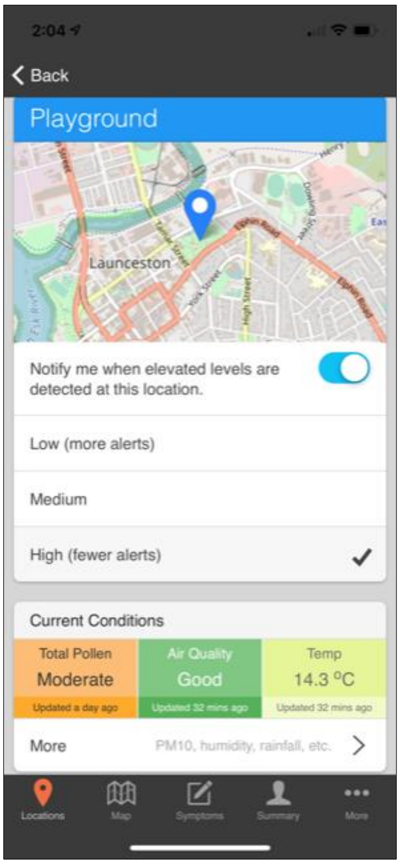

Level thresholds are set for each respective hazard based on the best available evidence for exposure-related health impacts in vulnerable populations. While many mHealth apps seek to address one specific medical condition or behavior, AirRater supports users suffering from multiple medical conditions, as well as those with no pre-existing medical conditions, to make informed decisions that protect health in the face of a variety of environmental hazards.

Table S2. Pre-interview questionnaire

Question No.	Question	Response options
**Opening statement**	Thank you for agreeing to take part in the evaluation of AirRater. Before you tell us about your experiences with AirRater in more detail in an interview or focus group discussion, it is important to know some information about you as a user and to gain a basic understanding of your thoughts about AirRater.<br><br>Below are some general questions about you. By answering the questions below, you will help us to better understand the different users of AirRater. The survey should take approximately 10 minutes to complete.<br><br>Your privacy is important to us. The information you provide below is anonymous. Any information you provide that we use for reporting will be grouped with information provided by all respondents so that you are not identifiable.<br><br>If you have any questions about this survey or for more information, please contact the AirRater team at <air.rater@utas.edu.au> or call 1800 322 102.	n/a
Your thoughts about AirRater	Q1. In your mind, what is the purpose of AirRater? (How would you describe AirRater to a friend?)	{Open text response}
	Q2. How did you find out about AirRater?	{Open text response}
	Q3. What was the main reason you decided to download AirRater?	{Open text response}
	Q4. Do you use AirRater for yourself, or do you use it for someone you care for? (e.g. a child with asthma)? (Please select one option only)	a. For myself<br>b. For someone I care for<br>c. I use it for my myself and for someone I care for
	Q5. Please provide a brief indication of how regularly you use AirRater.	{Open text response}

	Q6. Approximately how long ago did you download AirRater? (Please select one option only)	a. Less than 6 months ago<br>b. 6-12 months ago<br>c. 1-2 years ago<br>d. 2-3 years ago<br>e. 3-4 years ago<br>f. 4-5 years ago
Information about you	Q7. What is your gender? (Please select one option only)	a. Male<br>b. Female<br>c. Other<br>d. Prefer not to say
	Q8. What is your age range? (Please select one option only)	a.18-20<br>b. 21-30<br>c. 31-40<br>d. 41-50<br>e. 51-60<br>f. 61-70<br>g. 70+
	Q9. Do you currently experience any of the following medical conditions? (Please select all that apply)	a. Asthma<br>b. Lung condition other than asthma (chronic bronchitis, COPD or other)<br>c. Hay fever<br>d. Heart condition (heart failure, heart attack, angina)<br>e. Stroke or TIA<br>f. Diabetes<br>g. Pregnant<br>h. Other (please specify) {open text response}
	Q10. If you are a parent of a child/ren, what age is your child/ren? (Please select all that apply)	a. 0-5 years old<br>b. 5-10 years old<br>c. 10-15 years old<br>d. I am not a parent of a child in the three age brackets listed
	Q11. For Port Macquarie and ACT, were you pregnant during the smoky periods in your area between November 2019 and January 2020?	a. Yes<br>b. No<br>c. Not sure<br>d. I do not reside in Port Macquarie or ACT

	Q12. For Tasmania, were you pregnant and experienced smoky periods in your area between January and February 2019?	a. Yes<br>b. No<br>c. Not sure<br>d. I do not reside in Tasmania
	Q13. What is the postcode for the suburb where you reside?	{Open text response}
	Q14. What is your personal income bracket?	a. Nil income<br>b. $1-$10,399<br>c. $10,400-$15,599<br>d. $15,600-$20,799<br>e. $20,800-$31,199<br>f. $31,200-$41,599<br>g. $41,600-$51,199<br>h. $52,000-$64,999<br>i. $65,000-$77,999<br>j. $78,000-$103,999<br>k. $104,000 or more
Closing statement	Thank you for taking the time to complete the pre-interview/ focus group discussion questionnaire.<br>We look forward to hearing your thoughts on AirRater during an interview or focus group discussion soon.<br>If you have any questions, or need to confirm details for your participation in an interview or focus group, please contact the AirRater team at <air.rater@utas.edu.au> or call on 1800 332 102.	n/a

Table S3. Summary of key semi-structured interview questions, adapted from Workman et al (2021)

Primary question/prompt
**1. Use of AirRater**<br>Please tell me about your use of AirRater.
**2. Features of AirRater**<br>Please tell me about your experience with AirRater's features.
**3. Comprehension of and trust in AirRater**<br>Please tell me about your understanding of the information that AirRater provides.
**4. Self-management and AirRater**<br>Please tell me about whether you have used AirRater to support your health since you have downloaded the application.
**5. Behavior change and AirRater**<br>Please tell me about what you do once you have looked at the information provided by AirRater.
**6. Translational capacity of AirRater**<br>Please tell me about any discussions you have had about AirRater with other people.
**7. Anything else**<br>Is there anything that we have not discussed that you think might be relevant or of interest?

Table S4. Summary of participants' suggested enhancements to the app

**Suggested enhancements**
Additional data/information
More detailed breakdown of AQ/pollen data
Include wind speed/direction, air pressure, UV rating, humidity, other hazards e.g. VOCs
Health advice for different ratings and different populations
Include additional pollens beyond most common ones
Access to historical data on specific allergen, PM and pollen levels
Include an in-app explanation of relevant contextual information, e.g. what is air quality index, particulate matter, etc.
Enhancements to features and/or functionality
Automatically notify user of the nearest monitoring station/s
Include a predictive/forecast functionality
Support multiple profiles in the one device, ie. for multiple family members
Provide alerts for planned burns, specific pollen species and/or triggers, generic reminder to use the app
Include space for a general two-word comment in symptom reporting to capture experiences, e.g. asthma attacks
Support more accurate location sensitivity
Provide access to all personal data (not just last three months)
Provide ability to download personal summary data for specified time period
Compare personal data against environmental/weather factors e.g. humidity
Create an overlay for allergen 'hot spots'
When reporting symptoms, app should ask if user is indoors or outdoors
Send a prompt to 'add a location' where a pin has been dropped a few times
Include mental health in symptom reporting
Provide generic advice for different ratings, eg. for 'poor', close windows and doors
Allow users to minimize symptom reporting when they first open the app
Allow users to indicate they take certain medication daily
Allow users to tick two symptoms at the same time if symptoms are similar and meds are the same
Support app use overseas
Updates need to be more regular than hourly
Make map legend easily accessible
Allow graph overlays, e.g. personal symptoms, air quality data, etc.
Date and time stamp data and provide an indication of when the next data update is scheduled
Provide monthly averages for data in graphs to easily see patterns/trends and to compare with last year's data
Create accuracy rating for data readings that takes into account wind direction and speed
Clarification
Need to better explain what being a study participant involves
Clarify that a user's location cannot be searched by postcode
Provide more clarity around symptom reporting: are symptoms the result of an existing issues or is it a new issue due to environmental factors?
Integration
Link to other apps including 'Fires Near Me', 'AirRaterSmoke', 'Bureau of Meteorology', and 'WeatherZone'

Customization
Provide the ability to select which data/information is viewed in the app
Provide the ability to access personal trigger information on the home page
Other
Consider a study for people without symptoms
Provide greater incentives for app use
Communicate study results and/or public health data generated from app to users
